# Guanylin, Uroguanylin and Guanylate Cyclase-C Are Expressed in the Gastrointestinal Tract of Horses

**DOI:** 10.3389/fphys.2019.01237

**Published:** 2019-09-27

**Authors:** Katia Cappelli, Rodolfo Gialletti, Beniamino Tesei, Gabrio Bassotti, Katia Fettucciari, Stefano Capomaccio, Laura Bonfili, Massimiliano Cuccioloni, Anna Maria Eleuteri, Andrea Spaterna, Fulvio Laus

**Affiliations:** ^1^Department of Veterinary Medicine, University of Perugia, Perugia, Italy; ^2^School of Biosciences and Veterinary Medicine, University of Camerino, Camerino, Italy; ^3^Department of Medicine, School of Medicine, University of Perugia, Perugia, Italy; ^4^Department of Experimental Medicine, School of Medicine, University of Perugia, Perugia, Italy

**Keywords:** gastrointestinal tract, guanylate cyclase-C, guanylin, horse, uroguanylin

## Abstract

Guanylate cyclase-C (GC-C) is a multifunctional receptor encoded by the *GUCY2C* gene, representing an attractive target for therapy in several gastrointestinal diseases in humans. Little is known about this system in horses. We investigated for the first time the gene expression of guanylin, uroguanylin and GC-C receptors in different horse’s gastrointestinal tracts. Tissue samples from stomach, duodenum, jejunum, ileum, head and body of cecum, left and right dorsal colon, left and right ventral colon, pelvic flexure, transverse colon, descending colon and rectum were collected from adult horses within 1 h *post mortem*. For each sample, total RNA was extracted from 100 mg of ground tissue, and qRT-PCR performed on *GUCA2a*, *GUCA2b* and *GUCY2* transcripts on a CFX96 Touch instrument. Data analysis was carried out with Bio-Rad CFX Manager software, and genes of interest normalized relative to the abundance of the two reference genes (*SDHA, HPRT*). Additionally, the protein expression levels of GC-C receptor were analyzed through western blotting. A common pattern of expression throughout the gastrointestinal lumen for all three investigated transcripts was found. The expression of *GUCA2a*, *GUCA2b* and *GUCY2* genes was higher in jejunum, ileum, descending colon and rectum. The levels of expression of GC-C protein confirmed these data. The findings of this study might open new scenarios for the therapeutic approach to enteric diseases of horse using selective agonists of GC-C.

## Introduction

Guanylin and uroguanylin are endogenous hormones involved in intestinal fluid homeostasis and bowel function of the gastrointestinal tract in several species; these hormones activate the guanylate cyclase-C (GC-C), a type I transmembrane receptor essentially expressed on the apical surface of intestinal epithelial cells. Receptor activation by selective GC-C agonists, such as guanylin and uroguanylin, responds to several signals by catalyzing the conversion of guanosine triphosphate to cyclic guanosine-3′,5′ monophosphate (GMPc) (Lucas et al., 2000). The increase of intracellular concentrations of the GMPc second messenger triggers a signaling cascade that causes an uncontrolled release of electrolytes and water into the intestinal lumen, resulting in secretory diarrhea (Brierley, 2012). The intracellular GMPc accumulation proceeds to the PKGII-dependent (protein kinase II) phosphorylation of the cystic fibrosis transmembrane regulator (CFTR), stimulating transepithelial secretion of chloride and bicarbonate ions, and inhibition of sodium absorption by the sodium/hydrogen exchanger 3 (NHE3): these effects results in a net efflux of ions and water into the lumen (Forte, 1999; Steinbrecher and Cohen, 2011; Hannig et al., 2014; Ahsan et al., 2017).

Guanylate cyclase-C is also involved in mucosal barrier function, inflammation, modulation of intestinal cell proliferation, and pain sensation (Whitaker et al., 1997; Forte, 1999; Giannella and Mann, 2003; Blomain et al., 2016).

Guanylate cyclase-C is encoded by the *GUCY2C* gene, and represents one of seven mammalian transmembrane guanylate cyclase receptors (Forte, 1999; Danaee et al., 2017). Its transcript is mainly expressed on epithelial cells of the gastrointestinal tract and was at first identified as the receptor for the bacterial heat-stable enterotoxin (STa), produced by several enteric pathogens and responsible for the traveler’s diarrhea (Lin et al., 2010; Uranga et al., 2018).

Endogenous ligands of GC-C, guanylin and uroguanylin, are encoded by *GUCA2A* and *GUCA2B* genes. *GUCA2A, GUCA2B* and *GUCY2C* are down-regulated in human chronic inflammatory intestinal diseases with dysfunctional epithelial electrolyte regulation (Brenna et al., 2015). This increased knowledge on the activities triggered by GC-C activation has guided the development of novel therapeutic approaches to gastrointestinal disorders (Waldman and Camilleri, 2018), including constipation, which resulted in the clinical use of linaclotide, a synthetic 14-peptide, potent and selective agonist of GC-C and, more recently, of plecanatide and dolcanatide (Hannig et al., 2014; Shailubhai et al., 2015; Al-Salama and Syed, 2017; Laus et al., 2018).

Although several prokinetic drugs are administered to horses with gastrointestinal hypomotility in veterinary practice, their use and efficacy are not well established yet (Van Hoogmoed et al., 2004); among these, bethanecol and neostigmine are cholinomimetic drugs that can be administered to horses, with cholinergic side effects preventing their routine use (Sanchez, 2009) whereas the efficacy of metoclopramide and domperidone needs to be further investigated, irrespective of being proposed in the treatment of hypomotility, (Nieto et al., 2013). Other drugs with established prokinetic effect in horses include erythromycin, naloxone, and lidocaine in horses affected by post-operative ileus (Sanchez, 2009). Cisapride can be used in managing persistent large colon impaction in horses, equine grass sickness, and as a preventative for postoperative ileus. However, due to serious cardiac side effects in humans this drug has been withdrawn from the market (Quigley, 2011). A specific treatment for hypomotility in horse has therefore not yet been established, therefore the identification of drugs able to enhance the intestinal transit, relieve pain and soften the feces could be an extremely valuable tool in equine internal medicine.

In this context, in this paper, the expression of guanylin, uroguanylin and GC-C receptors, in different gastrointestinal tracts of horse has been investigated for the first time, with the aim of developing new therapeutic strategies for the treatment of gastrointestinal diseases in horses.

## Materials and Methods

### Animals and Samples Collection

Tissue samples were collected from six female, mixed breed horses (5–9 years old, mean age: 7.2 ± 1.5 SD). All animals had no history of gastrointestinal disorders and were slaughtered at a public abattoir. The Animal Care Committee of University of Camerino approved the study with authorization number E81AC.8/D.

Full-thickness samples were collected immediately *post mortem* from the stomach, duodenum, jejunum, ileum, head and body of cecum, left and right dorsal colon, left and right ventral colon, pelvic flexure, transverse colon, descending colon and rectum. All samples were immediately frozen in liquid nitrogen and then stored at –80°C.

### RNA Extraction

Total RNA of all samples was extracted from 100 mg of ground tissue using the Trizol Plus RNA purification kit (Ambion, Life Technologies, Monza, Italy) according to the manufacturer’s instructions, and checked for integrity and purity of nucleic acid as previously described (Brachelente et al., 2017; Stefanetti et al., 2017).

### qRT-PCR Assays

Total RNA (1 μg) of each sample was retrotranscripted using SuperScript^®^ IV Vilo Master Mix (Thermo Fisher Scientific, Rodano, Italy) according to the manufacturer’s specifications. In addition, 50 ng/μl of each sample were used to prepare a bulk solution for each gastrointestinal tract. Preliminary qRT-PCR was run on these bulk solutions to optimize experimental conditions and sub-select the most relevant gastrointestinal tracts, prior to the qRT-PCR assay on single samples.

Primers for the genes of interest (*GUCA2a*, *GUCA2b* and *GUCY2c*) were designed based on available sequences using the Primer-BLAST suite (Table 1); primers for reference genes (*SDHA* and *HPRT*) were those identified by Cappelli et al. (2008) and previously tested (Cappelli et al., 2009, 2013; Capomaccio et al., 2010, 2011). Primers for the gene of interest were designed taking into account all previously established requirements (Capomaccio et al., 2010, 2013; Brachelente et al., 2017). For each pair, a preliminary qRT-PCR assay was performed on the tissue bulks to check for amplification of either non-specific products or primer-dimer artifacts, and the efficiency were calculated. The qRT-PCR reaction was carried out aliquoting 5 μl of a five-fold diluted cDNA and SYBR Select Master Mix for CFX (Thermo Fisher Scientific). The amplifications were performed in a CFX96 Touch instrument (Bio-Rad, Hercules, CA), each sample was run in triplicate with appropriate negative controls, under the same condition used in previous studies: 98°C for 3 min, then 45 cycles of 98°C for 10 s and 60°C for 15 s. Fluorescence data were collected at the end of the second step and, following cycling, the melting curve was determined in the range of 58–95°C with an increment of 0.01°C/s (Brachelente et al., 2017).

**TABLE 1:** Primer combinations for the studied genes.

**Gene**	**Accession Number**	**Forward**	**Reverse**	**Amplicon size**	**Intron size**
*GUCA*	XM_0015031	CATGAACACCTTCC	AGGTCCTTGAGCT	126	1048
*2A*	67.2	TGCTCTCT	TCTTCACT		
*GUCA*	XM_0014975	GCTGTGGTCTTCCT	CCAGGTCACTCAG	103	1137
*2B*	86.2	AGTGCT	CTTCTTCA		
*GUCY*	XM_00150169	TCTTTTGACCTATG	TGCGAGAGTGAAG	142	5356
*2C*	8.3	ACACCCACA	ACTGCAA		

*Sizes are expressed in base pairs.*

### Data Analyses

Bio-Rad CFX Manager software (ver. 3.2.2) was utilized to analyze data, including reference genes stability through geNorm algorithm, available in the CFX Manager software (vers. 3.2.2) (Vandesompele et al., 2002). The expression ratio of the genes of interest was normalized relative to the abundance of the two reference genes using the ΔΔCq method. Samples for each gene of interest were grouped as biological replicates (one bulk or 6 horses) and tissues (14 tissues or 6 tissues), and analyzed accordingly. Expression levels are presented as mean values with standard error in a base 2 logarithmic scale or in relative expression, the least expressed condition being selected as baseline. We exported qRT-fPCR data for the 6 horses group after normalization with reference genes, and performed analysis of variance (ANOVA) using Tukey–Kramer *post hoc* test for the comparison of mean values.

### Western Blotting Analyses

Tissues were homogenized in 50 mM Tris buffer, 150 mM KCl, 2 mM EDTA, pH 7.5 (1:5 weight/volume) and centrifuged at 13.000 × *g* for 20 min (4°C). Protein content was determined by the Bradford method (Bradford, 1976) using bovine serum albumin (BSA) as standard. Tissues homogenates were analyzed through western blotting assays with the aim to measure guanylyl cyclase protein expression levels. In detail, for each sample 20 μg total proteins were loaded on 10% SDS-PAGE and electroblotted onto PVDF membranes. Successively, upon incubation with the specific antibody Anti-*GUCY2C* (ab213430, Abcam, Italy, Supplementary Figure S2) the immunoblot detections were carried out through Enhanced Chemiluminescence (ECL) western blotting analysis system (Amersham Pharmacia-Biotech). β-actin was used to check equal protein loading, stripping the membranes and incubating with the anti-β-actin primary antibody (Santa Cruz Biotechnology, Italy). The bands were quantified by using a previously described densitometric algorithm (Schneider et al., 2012). Statistical analysis was executed using one-way ANOVA, followed by the Bonferroni *post hoc* test using Sigma-stat 3.1 software (SPSS, Chicago, IL, United States). *P* values < 0.05 were considered to be significant.

### Protein Sequence Alignment

Guanylate cyclase-C amino acid sequences from different species were obtained from UniProt (UniProt Consortium, 2019), and alignments and percent identity matrix analysis were performed using Clustal Omega (Sievers et al., 2011) ver. 2.1, with all settings kept to default.

UniProt accession numbers of protein sequences used are: *Equus caballus*: A0A3Q2L9C5; *Monodelphis domestica*: F7B751; *Gorilla gorilla gorilla*: G3R542; *Capra hircus*: A0A452E207; *Macaca fascicularis*: A0A2K5TZ15; *Ailuropoda melanoleuca*: G1LVN9; *Bos taurus*: F1N5B2; *Vulpes vulpes*: A0A3Q7S8U5; *Ornithorhynchus anatinus*: F6W2A3; *Cavia porcellus*: H0VCJ2; *Homo sapiens*: P25092.

### Prediction of Three-Dimensional Structures of Human and Horse GC-C

P25092 and A0A3Q2L9C5 UniProt entry sequences were used to model three dimensional structures of human and horse GC-C, respectively. Fold-recognition modeling was performed using I-Tasser (Yang et al., 2015), the best structural templates being 4PE5A, 5IJOJ, 5YFPB, 5VCHA, 5UOWB, 4UQQA, 5DLQB, 5YFPD, 4R04A, 6R3QA, and 6R3QA, 5OYHA, 4R04A, 5O5LA, 5IJOJ, respectively for human and horse. Structure refinement of the predicted models was carried out using ModRefiner (Xu and Zhang, 2011). Energy minimization of output models was performed with GROMOS 96 forcefield (Van Gunsteren, 1996), and models of GC-C were eventually submitted to PROCHECK (Laskowski et al., 2001) for backbone structure validation. Transmembrane domains were predicted using TMHMM Server v. 2.0 (Krogh et al., 2001).

### Docking Analysis of Linaclotide to GC-C

The predictive models of the complexes between human or horse GC-C and linaclotide (retrieved from PubChem: (Kim et al., 2019) was computed by docking the drug to the fold-recognition models of the protein. Rigid docking was performed using PatchDock server (Schneidman-Duhovny et al., 2005), human or horse GC-C and linaclotide being uploaded as receptor and ligand, respectively. FireDock was used for interaction refinement. Settings were always kept to default values (Andrusier et al., 2007; Mashiach et al., 2008). The best scoring complex and all images were rendered with PyMOL (The PyMOL Molecular Graphics System, Version 1.3 Schrödinger, LLC).

## Results

**FIGURE 1:**
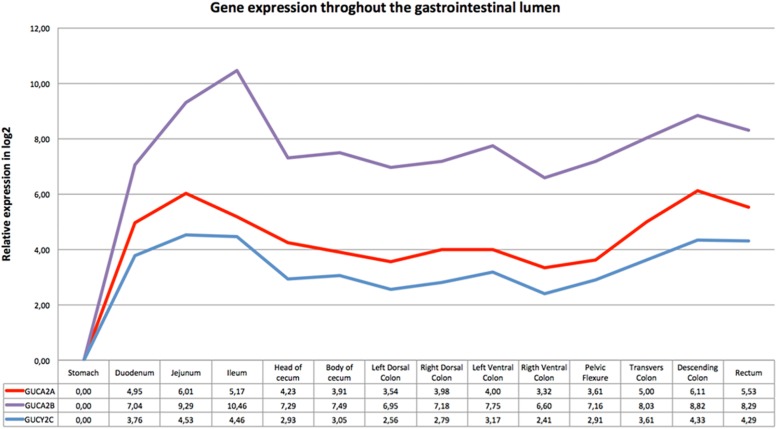
Gene expression throughout the gastrointestinal tract: the *x*-axis shows the different intestinal segments; the relative expression in log2 normalized for the two reference genes is indicated below the graph. The stomach, tissue with the lowest expression of all three genes, was set as calibrator. In the *y*-axis is indicated the relative expression in log2 scale.

Figure 1 shows the relative expression distribution of guanylin (*GUCA2A*), uroguanylin (*GUCA2B*) and guanylate cyclase-C (*GUCY2C*) transcripts in 14 regions of the horse gastrointestinal tracts. The expression values are normalized with two reference genes (*SDHA* and *HPRT*). Reference genes showed relatively high stability with M value of 0.23, far below the accepted threshold as stated by Vandesompele et al. (2002). A common pattern of expression was observed throughout the gastrointestinal tract for all three investigated transcripts. The expression was higher in jejunum, ileum, descending colon and rectum, and lower in the stomach.

**FIGURE 2:**
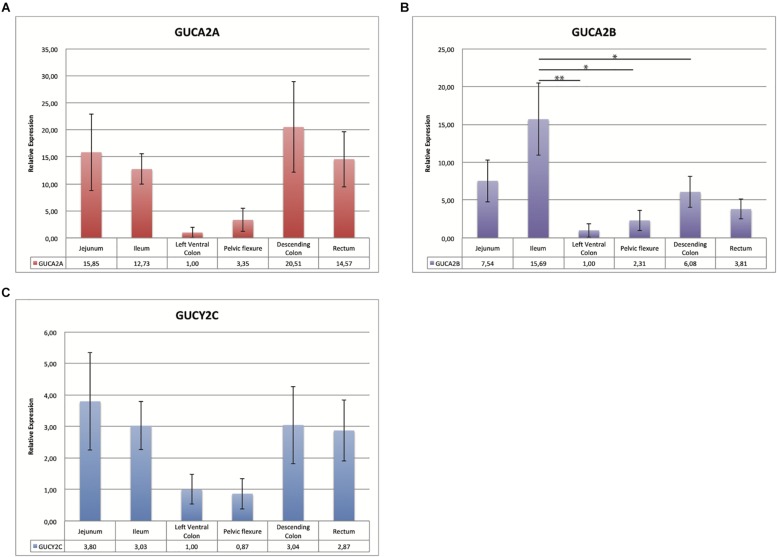
Bar charts representing the relative expression value of *GUCA2A* **(A)** *GUCA2B* **(B)** and *GUCYC2* **(C)**: the *x*-axis shows six different intestinal tracts; the relative expression for the six horses for each of the 3 genes tested, normalized for the two reference genes, is also indicated below the graph. The left ventral colon, tissue with the lowest expression, was set as calibrator. In the *y*-axis is indicated the relative expression value with standard deviation. Bars marked with an asterisk indicate significant statistical difference (^∗^*p* < 0.05 and ^∗∗^*p* < 0.001).

qRT-PCR results from bulk sample analyses (Figure 1) allowed us to reduce from 14 to 6 gastrointestinal tracts the gene expression analysis. These six tissues were further investigated using individual samples (Figure 2) to evaluate the individual variability that is lost with bulk solutions.

As shown in Figures 2A–C, the expression levels in tissues from six horses were comparable in bulk and individual samples. The individual variability is indicated by the values of standard error. The same expression pattern was observed with the highly expressed tissue ranging from 10 to 20 times compared to the calibrator for *GUCA2A*, from 3 to 15 for *GUCA2B* and of about 3 for *GUCYC2*. Difference in relative expression between genes can be seen in Figure 1, the pelvic flexure behaving like the other tissues in terms of pattern and having mild relative expression values.

Changes in the relative expression for the three genes were checked for statistical significance with ANOVA. Figure 2 shows that statistically significant differences are reached only for *GUCA2B* in the highlighted comparisons.

**FIGURE 3:**
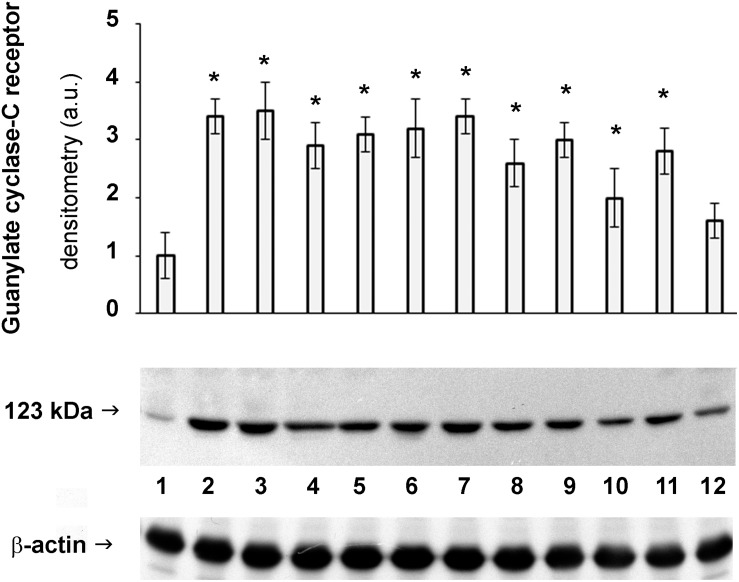
Protein expression of GC-C receptor in horse intestine. Detection of the levels of *GUCY2C* in stomach (1), duodenum (2), jejunum (3), ileum (4), body of cecum (5), left and right dorsal colon (6 and 7, respectively), left ventral colon (8), pelvic flexure (9), transverse colon (10), descending colon (11) and rectum (12). The densitometric analysis derives from six separate blots and a representative immunoblot is shown. Equal protein loading was verified by using an anti β-actin antibody. The detection was executed by an ECL western blotting analysis system. Data points marked with an asterisk indicate statistically significant differences with respect to the stomach, tissue with the lowest levels of GC receptor (^∗^*p* < 0.05).

Being GC-C receptor a promising therapeutic target, its protein expression levels were measured through western blotting. Results reported in Figure 3 confirm the expression pattern above described for the GC-C receptor.

### Protein Alignment

**FIGURE 4:**
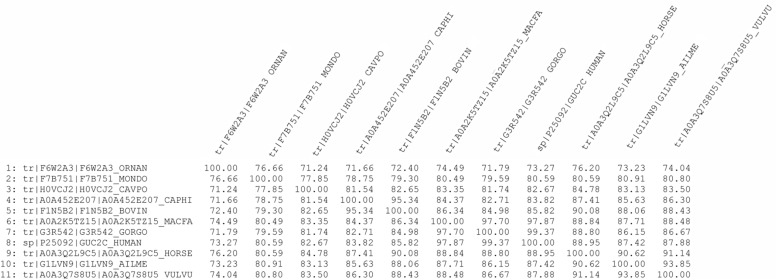
Percent identity matrix of GC-C amino acid sequences from different mammals using Clustal Omega.

Sequence alignment using Clustal Omega showed that GC-C is highly conserved among species (see percent identity matrix, Figure 4). Most interestingly, 86 and 88% identity, along with 98 and 94% high consensus homology, were observed in the guanylate cyclase and protein kinase catalytic domains (Supplementary Figure S1).

### Docking Analysis of Linaclotide to Guanylate Cyclase

**FIGURE 5:**
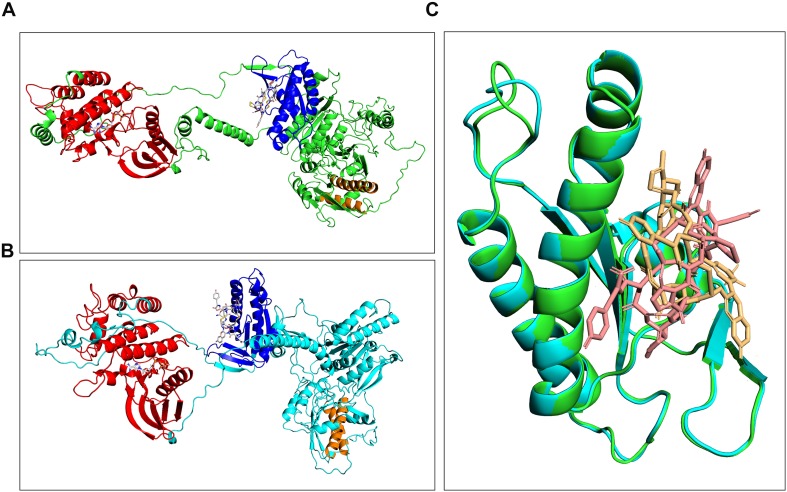
Comparison of complexes formed between either horse **(A)** or human GC-C **(B)** and linaclotide obtained by molecular docking. Cyclase and kinase catalytic domains are highlighted as blue and red ribbon, and corresponding binding regions are traced by linaclotide and GTP, respectively. Predicted transmembrane domains are highlighted in orange. Superimposition of the catalytic domains of human and horse guanylate cyclase complexed with linaclotide **(C)**.

The high homology between human and horse GC-C (particularly in the case of the catalytic domains) was associated to highly conserved three-dimensional structures (Figures 5A,B). Most interestingly, docking studies between the fold-recognition models of GC-C and linaclotide revealed a fully conserved binding mode between the ligand and the receptors, as evident from the superimposition of the complexes (Figure 5C). Specifically, the guanylate cyclase catalytic domain was calculated to be the most likely to accommodate the linaclotide molecule. This common behavior suggested a similar effect of the drug both in humans and horses.

## Discussion

The activation of the GC-C/cGMP pathway *via* guanylin and uroguanylin has been well established in various species; this pathway was shown to be essential in the restoration of mucosal barrier function during intestinal disorders, and the administration of synthetic agonists of this cascade can have beneficial effects. Being an essential prerequisite for their use, organ expression of *GUCA2A*, *GUCA2B* and *GUCYC2* has been investigated in previous studies in laboratory animals and humans (Wiegand et al., 1992; Hamra et al., 1996; Forte, 1999).

In the present study we found high expression of both mRNAs of *GUCA2A* and *GUCA2B*, with uroguanylin generally displaying higher levels with respect to guanylin. The highest expression was found in jejunum and ileum, whereas lower levels were observed in the small colon (descending colon), left ventral colon (ascending colon), and rectum. From these results, we can reasonably state that horse stomach has the lowest expression of guanylin mRNA as already established in other species such as opossum and humans.

Similarly to other animals, the highest expression level was detected in the ileum, whereas horses showed a high expression in the colon comparable to humans, rats and mice but different from the opossum (Wiegand et al., 1992; Hamra et al., 1996; Forte, 1999). Concerning uroguanylin, horses had higher expression in the large compared to the small intestine (upper tract), differently from other laboratory animals but similarly to the opossum (Kita et al., 1994; Hamra et al., 1996; Forte, 1999).

Our data suggest that outcomes from clinical studies for the activity of guanylin in the different gastrointestinal tracts of laboratory animals can be translated to the equine species; however, caution should be used regarding uroguanylin.

The expression pattern for *GUCY2C*, the gene encoding for the GC-C receptor, was highest in the jejunum, ileum, left ventral colon, descending colon and rectum nevertheless the large individual variability did not allow to achieve statistical significance. These findings are of considerable clinical interest, since the distal tract of the small intestine and the large intestine are more frequently affected by constipation in this species (Blikslager, 2010). Orally administered linaclotide, a selective agonist of GC-C, elicited a significant, dose-dependent increase in gastrointestinal transit rates in rats (Busby et al., 2010). This drug is currently administered orally in humans for the treatment of irritable bowel syndrome (IBS) with constipation and for chronic constipation, due to its ability to stimulate fluid secretion and accelerating transit (Andresen et al., 2007; Lembo et al., 2011; Bassotti and Blandizzi, 2014; Hannig et al., 2014). Interestingly, linaclotide can also induce peripheral visceral analgesia by activation of a GC-C/extracellular cGMP pathway (Hannig et al., 2014). Recently, a novel synthetic analog of human uroguanylin, plecanatide, received its first approval in the United States for the treatment of adult patients with chronic idiopathic constipation and IBS with constipation (Al-Salama and Syed, 2017; Miner, 2018). Future clinical trials in horses are warranted to understand biochemical implication of the variance in expression of the GC-C ligands across the gut.

Colonic impaction is a frequent cause of colic in horses, and its medical treatment may include several options such as oral and intravenous administration of fluids, laxatives, mineral oil and drugs for the control of visceral pain (Hanson, 2002). The use of common prokinetic drugs to treat horses with colonic impactions is controversial, because of the risk for intestinal rupture and pain worsening (Hanson, 2002). A recent study investigated an alternative approach to horse colonic impaction and reported the preliminary positive effects on motility of prucalopride, a drug acting as high-affinity agonist of serotonin receptors (5-HT4) (Laus et al., 2017). However, the activation of GC-C receptors on horse epithelial intestinal surface could lead to four simultaneous actions (e.g., softening of the feces, transit acceleration, pain relieving and mucosal barrier restoration) resulting in a more proficient and safer therapeutic approach to this condition.

Motility enhancing and transit improving drugs have been advocated to treat postoperative ileus (POI) in horse (Sanchez, 2009). The prevalence of POI is as high as 53% and it is the reason for euthanasia or death for up to 43% of horses that undergone abdominal surgery (Salem et al., 2016). Since recent preclinical studies suggest the use of linaclotide for treatment of POI in human (Uranga et al., 2018), the present demonstration of GC-C receptors in horses, together with the high identity/homology of GC-C catalytic domains among species (Figure 4) could open new scenarios to treat this severe condition in horses and other species (Supplementary Figure S1).

Pharmacological activation of GC-C could also be of benefit in duodenitis-proximal jejunitis, grass sickness and other hypomotility condition where prokinetics are indicated (Javsicas, 2009; Sanchez, 2009; Laus et al., 2017).

Drugs able to accelerate transit could also shorten the length of hospitalization of sick horses, thereby reducing the cost of treatment and the number of potential complications, such as weight loss, thrombophlebitis, and laminitis in case of the abovementioned diseases (Sanchez, 2009). Unfortunately, specific information pertinent to equines is limited and must be extrapolated prudently from other species, included humans, because of the important differences in anatomy and physiology (Sanchez, 2009).

Thus, the demonstration of gene expression pattern of guanylin, uroguanylin and GC-C receptors in the horse intestine could open new ways for the therapeutic approach to horse gastrointestinal diseases by GC-C agonist, offering the advantage of accelerating transit together with pain control. Acting directly on the epithelial surface, GC-C agonists are little or not at all adsorbed in the blood, limiting the possibility of side effect in other organs (Uranga et al., 2018). This is not the case of all other drugs currently available. Furthermore, GC-C synthetic agonists have the advantage to be administered on a daily basis. This is particularly useful in inappetent animals in which the drug could be administrated by nasogastric intubation. Donkeys are normally treated like small horses but they have many peculiarities in incidence, presentation and treatment of diseases (Laus et al., 2010). Nevertheless, since they have similar gastrointestinal physiology (herbivorous but monogastric) and can be affected by diseases causing life-threatening hypomotility (e.g., hyperlipidemia), the development of these kind of drugs could give advantages also in the management of these animals.

Interestingly, the receptor GC-C has been also demonstrated as a tumor suppressor, and has emerged as a promising target for intestinal and pancreatic cancer (Kloeters et al., 2008; Aka et al., 2017). The identification of GC-C receptors in horses as well as, in future, in other domestic species commonly affected by this type of cancer, could be an interesting field of study for new conservative therapies to be used in veterinary medicine.

## Conclusion

This study demonstrated for the first time the expression of genes encoding for guanylin, uroguanylin and GC-C receptor in the intestinal tract of horses. The highest expression levels were found in the distal tract of the small intestine and in the large intestine. These findings might open new scenarios for translational therapeutic approach to some enteric diseases in this species. In fact, available bioinformatics tools can be of great help in assessing the inter-species structural homology of pharmacological targets of interest. Case-in-point, the computational analysis of conserved amino acid residues of GC-C receptor and its binding to linaclotide provided a solid rationale (in terms of drug-receptor interaction) supporting its therapeutic use. Studies further exploring these possibilities are currently in progress.

## Data Availability Statement

The datasets generated for this study are available on request to the corresponding author.

## Ethics Statement

This study was carried out in accordance with the recommendations of European directive 2010/63/EU. The protocol was approved by the “Committee for protection of animals used in scientific research, University of Camerino.” This study was carried out on sampled collected on a public abattoir and no live animal have been included in the research.

## Author Contributions

KC, RG, SC, MC, and LB performed the laboratory investigations and analyzed the data. BT, KF, AE, and AS performed the laboratory investigations and gave important intellectual contributions. GB and FL conceived and designed the research, performed the experiments, and wrote the first draft of the manuscript. FL procured the animal samples. All authors reviewed and approved the final version of the manuscript.

## Conflict of Interest

The authors declare that the research was conducted in the absence of any commercial or financial relationships that could be construed as a potential conflict of interest.

## Supplementary Material

FIGURE S1 Alignment of guanylate cyclase protein sequences using ClustalW2.

FIGURE S2 Epitope sequences alignment on ClustalW of several species.
