# Supplementary material for: Guanylin, Uroguanylin and Guanylate Cyclase-C Are Expressed in the Gastrointestinal Tract of Horses
Source: Front Physiol. 2019 Sep 27;10:1237. doi: 10.3389/fphys.2019.01237 (PMC6776823; doi:10.3389/fphys.2019.01237)
Supplement: FIGURE S1 — Alignment of guanylate cyclase protein sequences using ClustalW2. [file Image_1.pdf]

```
TR|A0A3Q2L9C5|A0A3Q2L9C5_HORSE ------------------------------------------------------------
TR|A0A452E207|A0A452E207_CAPHI -------------------------------------------MKSPLLALALWSLL--- 14
TR|A0A2K5TZ15|A0A2K5TZ15_MACFA ------------------------------------------------------------
TR|G3R542|G3R542_GORGO         -------------------------------------------MKTLLLDLALWSLL--- 14
TR|G1LVN9|G1LVN9_AILME         -------------------------------------------TKMTLLGLALWSLL--- 14
TR|F1N5B2|F1N5B2_BOVIN         -------------------------------------------MKTPLLALALWSLI--- 14
TR|A0A3Q7S8U5|A0A3Q7S8U5_VULVU MDLRQLCQLESVLAKTIEVLGGKNCAQNKTSEHKEYGSEVSKVLKMILLGLALWSLL--- 57
TR|F6W2A3|F6W2A3_ORNAN         -------------------------------------------MEGLGLGVALGTLLL-- 15
TR|H0VCJ2|H0VCJ2_CAVPO         -------------------------------------------MKSPLLGLVVWSLLLQL 17
SP|P25092|GUC2C_HUMAN          -------------------------------------------MKTLLLDLALWSLL--- 14
TR|F7B751|F7B751_MONDO         ------------------------------------------------------------


TR|A0A3Q2L9C5|A0A3Q2L9C5_HORSE ------------------------------------------------------------
TR|A0A452E207|A0A452E207_CAPHI LRPGLPFWTSHVSRNCQDGSYEISVLMMNNSAFPEFLDSLEEVVKEGVKIVRQRLLE--- 71
TR|A0A2K5TZ15|A0A2K5TZ15_MACFA --------------------------MMDNSAFAEPLENVEDAVNEGLEIVRGRLQN--- 31
TR|G3R542|G3R542_GORGO         FQPRWLSLSSKVSQNCHNGSYEISVLMMDNSAFAEPLKNLEDAVNEGLEIVRGRLQN--- 71
TR|G1LVN9|G1LVN9_AILME         LCPGLTFWDSQISRNCNNGSYEISVLMMNNSAFPESLDNLKEAVNEGVEIVRQRLLNAVL 74
TR|F1N5B2|F1N5B2_BOVIN         LRPGLPFWTSHVSRNCQDGSYEISVLMMNNSAFPESLDSLEEVVKEGVKIVSQRLLE--- 71
TR|A0A3Q7S8U5|A0A3Q7S8U5_VULVU LRPGVMYWASQISQNCNNGSYEISVLMMNNSAFPESLDNLKEAVNEGVEIVRQRLLN--- 114
TR|F6W2A3|F6W2A3_ORNAN         -GFQVLGWATTGNPHCRKDFYELSVLMMSSSAFPESLKLVRPAVELGVKLVEELLTES-- 72
TR|H0VCJ2|H0VCJ2_CAVPO         LQPGLAFWNSQISQNCHNGSYEITVLMMNNYAFQESLESLKTAVNKGLDIVKQRLQE--- 74
SP|P25092|GUC2C_HUMAN          FQPGWLSFSSQVSQNCHNGSYEISVLMMGNSAFAEPLKNLEDAVNEGLEIVRGRLQN--- 71
TR|F7B751|F7B751_MONDO         ------------------------------------------------------------


TR|A0A3Q2L9C5|A0A3Q2L9C5_HORSE --------------------------------------------MGCALMGPSCTYSTFQ 16
TR|A0A452E207|A0A452E207_CAPHI -AGLNVTVNATFIYSEGVIYKSNDCRSSTCEGLDLLRTISSKKQMGCVLMGPSCTYSTFQ 130
TR|A0A2K5TZ15|A0A2K5TZ15_MACFA -AGLNVTVNASFMYSDGLIHNSGDCRSSTCEGLDLLRKISNAKRMGCVLMGPSCTYSTFQ 90
TR|G3R542|G3R542_GORGO         -AGLNVTVNATFTYSDGLIHNSGDCRSSTCEGLDLLRKISNAQRMGCVLIGPSCTYSTFQ 130
TR|G1LVN9|G1LVN9_AILME         ETGINVTVNVTFIYSDSVIYKSNDCRSSTCEGLDLLRAISNNKRMGCVLMGPSCTYSTFQ 134
TR|F1N5B2|F1N5B2_BOVIN         -AGLNVTVNATFIYSEGVIYKSSDCRSSTCEGLDLLRTISSKKQMGCVLMGPSCTYSTFQ 130
TR|A0A3Q7S8U5|A0A3Q7S8U5_VULVU -AGKNVTVNATFIYSNTVIYKSSDCRSSTCEGIDLLREISNNKRMGCVLMGPSCTYSTFQ 173
TR|F6W2A3|F6W2A3_ORNAN         ESGHDVQVNASFFESESPIYRSTDCRSSTCK-LQIFMDIEKGKKMGCALMGPSCTYATFQ 131
TR|H0VCJ2|H0VCJ2_CAVPO         -AALYVTVNATFIHSDGLIHKSGDCRSSTCEGLDLLREIARQKRMGCALMGPSCTYSTYQ 133
SP|P25092|GUC2C_HUMAN          -AGLNVTVNATFMYSDGLIHNSGDCRSSTCEGLDLLRKISNAQRMGCVLIGPSCTYSTFQ 130
TR|F7B751|F7B751_MONDO         -------------------------------------------------MGPSCTYSTYQ 11
                                                                                :******:*:*

TR|A0A3Q2L9C5|A0A3Q2L9C5_HORSE MYLDTDLNYPMISAGSFGLSCDYKETLIRLMSPARKLMYFLVNFWKANNLPFKTFSWSTA 76
TR|A0A452E207|A0A452E207_CAPHI MYLDTDLNYPMISAGSFGLSCDYKETLTRMMSPARKLMYFLVDFWKG-NFQFKPFPWNTA 189
TR|A0A2K5TZ15|A0A2K5TZ15_MACFA MYLDTELSYPMISAGSFGLSCDYKETLTRLMSPARKLTYFLVNFWKTNDLPFKTYSWSTS 150
TR|G3R542|G3R542_GORGO         MYLDTELSYPMISAGSFGLSCDYKETLTRLMSPARKLMYFLVNFWKTNNLPFKTYSWSTS 190
TR|G1LVN9|G1LVN9_AILME         MYLDADLNYPMISAGSFGLSCDYKETLTRLMSPARKLMYFLVNFWKVNDLPFKSFSWNSA 194
TR|F1N5B2|F1N5B2_BOVIN         MYLDTDLNYPMISAGSFGLSCDYKETLTRMMSPARKLMYFLVEFWKV-KFQFKPFSWNTA 189
TR|A0A3Q7S8U5|A0A3Q7S8U5_VULVU MYLDTDLNYPMISAGSFGLSCDYKETLTRLMSPARKLMYFLVDFWKANNLPFKDYSWKTA 233
TR|F6W2A3|F6W2A3_ORNAN         MYLDMELDLPTISAGSFGLSCDFKETLTRLMTPARKLTNFLVDFWKMSNLPFKTTPWKTS 191
TR|H0VCJ2|H0VCJ2_CAVPO         MYLDTELNYPMISAGSFGLSCDYKETLTRMMSPARKLMYFLVDFWKASNLPFKSFSWNTS 193
SP|P25092|GUC2C_HUMAN          MYLDTELSYPMISAGSFGLSCDYKETLTRLMSPARKLMYFLVNFWKTNDLPFKTYSWSTS 190
TR|F7B751|F7B751_MONDO         MYLDTILEYPMISAGSFGLSCDHKETLTRLMFPARKLTYFLVDFWKTHNLTFKTVSWKTA 71
                               ****  *. * ***********.**** *:* *****  ***:***  .: **   *.::

TR|A0A3Q2L9C5|A0A3Q2L9C5_HORSE YVFKNGSESEDCFWYLNALEAGVSYFSQELIFKDVLRGDDQFQGILSNHKRKSNVILMCG 136
TR|A0A452E207|A0A452E207_CAPHI YVFKNSTETEDCFWYLNALEAGVSYFSQNLGFKEMLRGDTEFQDILMDQNRKSNVIVMCG 249
TR|A0A2K5TZ15|A0A2K5TZ15_MACFA YVYKNGTESEDCFWYLNALEASVSYFSHELSFKLVLRQDKEFQDILMDHNRKSNVIVMCG 210
TR|G3R542|G3R542_GORGO         YVYKNGTETEDCFWYLNALEASVSYFSHELGFKVVLRQDKEFQDILMDHNRKSNVIIMCG 250
TR|G1LVN9|G1LVN9_AILME         YVFKNGSETEDCFWYLNALEAGVSYFSQELSFKEMLRGNDQFQDILTNQNRKSNVIVMCG 254
TR|F1N5B2|F1N5B2_BOVIN         YVFKNSTETEDCFWYLNALEAGVSYFSQKLGFKEMLRGDTEFQDILMDQNRKSNVIVMCG 249
TR|A0A3Q7S8U5|A0A3Q7S8U5_VULVU YVFKNSTESEDCFWYLNALEAGVSYFSQELSFKEMLRGDDQFQDILMNQNRKSNVIVTCG 293
TR|F6W2A3|F6W2A3_ORNAN         YVYKNQNLSEDCFWYLNALEAGVTYFSGILEFKEVLRNEEQFQAFLTDQNRKSNVIIMCG 251
TR|H0VCJ2|H0VCJ2_CAVPO         YVFKNGTESEHCFWYINALEAGVSYFSQVLGFKEMLRGNEELQKILKDPNRRSNVIVMCG 253
SP|P25092|GUC2C_HUMAN          YVYKNGTETEDCFWYLNALEASVSYFSHELGFKVVLRQDKEFQDILMDHNRKSNVIIMCG 250
TR|F7B751|F7B751_MONDO         YVFKNNNYTEECFWYLNALEAGVSYFSQELQFKDILRTEEQFQNILTNKNRKSNVIVMCG 131
                               **:** . :*.****:*****.*:***  * ** :** : ::* :* : :*:****: **

TR|A0A3Q2L9C5|A0A3Q2L9C5_HORSE RPDVIHSLKGDRAVAEDIVIILVDLFNN-HYFTDNVTAPDYMKNVLVLTLPPENSTSNGS 195
TR|A0A452E207|A0A452E207_CAPHI RPETIRNLRELSFQKENKDFQK--IFSF-LMFF--------------IA---LNG--IQD 287
TR|A0A2K5TZ15|A0A2K5TZ15_MACFA DPQFLYKLKGDRAVAEDIVIILVDLFND-QYFEDNVTAPDYMKNVLVLTQSPGNSLLNSS 269
TR|G3R542|G3R542_GORGO         GPEFLYKLKGDRAVAEDIVIILVDLFND-QYFEDNVTAPDYMKNVLVLTLSPGNSLLNSS 309
TR|G1LVN9|G1LVN9_AILME         TPSVISNLKGDRAVAEDIVIILVDLFNN-HYFMENVTAPDYMKNVLVLTLPPENSTSISS 313
TR|F1N5B2|F1N5B2_BOVIN         RPETIQNLRGNRAVAEDIVIILVDLFND-HYFMDNVTAPDYMKNVLVLTLPPENSVSNSS 308
TR|A0A3Q7S8U5|A0A3Q7S8U5_VULVU TPSTISSLKGDRAVAEDIVIILVDLFNN-DYFMDNVTAPNYMKNVLVLTLPPEYSISNSS 352
TR|F6W2A3|F6W2A3_ORNAN         TPEIVSEIVGGRQVDEDIIIILVDLFSSSKYFEDPKQSADYMHNVLVVTLPPPPVMSSVV 311
TR|H0VCJ2|H0VCJ2_CAVPO         TPQTMESLKIDWTATEDTVIILVDLFNN-YYFEENATAPDYMKNVLVLTLPPGNSTINTS 312
SP|P25092|GUC2C_HUMAN          GPEFLYKLKGDRAVAEDIVIILVDLFND-QYFEDNVTAPDYMKNVLVLTLSPGNSLLNSS 309
TR|F7B751|F7B751_MONDO         SPESVNALKGDHESDEDIVIILVDLFNN-QYFENTT-SAPYMKNVLVVTLMPANSSLSNL 189
                                *. :  :       *:  :    :*.    *               ::
```

```
TR|A0A3Q2L9C5|A0A3Q2L9C5_HORSE FFKDFSLAKNDFSLAYLDGVLLFGHMLKIFLESGEDVTTPKFAHAFRNLTFEGYTGPVTL 255
TR|A0A452E207|A0A452E207_CAPHI KDNIVTRAKNDFAAAYLDGVLLFGHMLKIFLENGEDVTTSKFAHAFRNITFEGHVGPVTL 347
TR|A0A2K5TZ15|A0A2K5TZ15_MACFA FSRNLSPTKRDFALAYLNGILLFGHMLKTFLENGENITTPKFAHAFRNLTFEGYDGPVTL 329
TR|G3R542|G3R542_GORGO         FSRNLSPTKRDFALAYLNGILLFGHMLKIFLENGENITTPKFAHAFRNLTFEGYDGPVTL 369
TR|G1LVN9|G1LVN9_AILME         FSKGLSQAKNNFALAYLNGILLFGHMLKIFLENGEAITTPKFAQAFRNLTFEGHAGPVTL 373
TR|F1N5B2|F1N5B2_BOVIN         SSKNLSLAKNDFAAAYLDGVLLFGHMLKIFLENGEDVTTSKFAHAFRNITFEGHVGPVTL 368
TR|A0A3Q7S8U5|A0A3Q7S8U5_VULVU FSKGTPQAKNNFALAYLNGVLLFGHMLKTFLENGEAITTPKFAQGFRNLTFEGHAGPVTL 412
TR|F6W2A3|F6W2A3_ORNAN         LESGSVQLKDEFAVAYLDGVLLFGHMLKKLLQNKETL-DFSFGRLFRNLTFPGALGPVTV 370
TR|H0VCJ2|H0VCJ2_CAVPO         LSKESLQEFSDFALAYLDGILLFGHMLKTFLRNGENTTAHKFAHAFRNLTFEGSTGPVTL 372
SP|P25092|GUC2C_HUMAN          FSRNLSPTKRDFALAYLNGILLFGHMLKIFLENGENITTPKFAHAFRNLTFEGYDGPVTL 369
TR|F7B751|F7B751_MONDO         --LNISLVKDDFVLAHLDGILLFGHMVKKSLHDN---SSAYFSHAFRNLTFQGALGPVTL 244
                                         :*  *:*:*:******:*  *..        *.: ***:** *  ****:

TR|A0A3Q2L9C5|A0A3Q2L9C5_HORSE DDCGDIDNTMMLLYTSVETNKYKVLLTYDTHINGTKPVDMSPTFMWKNHKLPNDIPGQGP 315
TR|A0A452E207|A0A452E207_CAPHI DACGDIDNTMYLLYTSVDTSKYKVLLTYDTRVNQTSPVDKSPTFIWKNHKLPNDIPGRGP 407
TR|A0A2K5TZ15|A0A2K5TZ15_MACFA DDWGDVDSTMVLLYTSVDTKKYKVLLTYDTHVNQTNPVDMSPTFTWKNSKLPNDITDRGP 389
TR|G3R542|G3R542_GORGO         DDWGDVDSTMVLLYTSVDTKKYKVLLTYDTHVNKTYPVDMSPTFTWKNSKLPNDITGRGP 429
TR|G1LVN9|G1LVN9_AILME         DDCGDIDNTMVLLYTSVETNKYKVLLKYDTHVNKTTPEVDNPMFIWMNHKLPSDIPGQGP 433
TR|F1N5B2|F1N5B2_BOVIN         DACGDIDNTMYLLYTSVDTSKYKVLLTYDTRVNQTSPVDKSPTFIWKNHKLPNDIPGQGP 428
TR|A0A3Q7S8U5|A0A3Q7S8U5_VULVU DDCGDIDNTMMLLYTSVDTNKYKVLLTYDTHINKTTRVDKRPTFIWMNHKLPNDIPGQGP 472
TR|F6W2A3|F6W2A3_ORNAN         DEFGDVDSNMTLLYTSMGNNKYKVLMHYDTHQNKSYPRDPDPTFIWKKGKLPSDKPGQGL 430
TR|H0VCJ2|H0VCJ2_CAVPO         DDSGDIDNTMVLLYTSVDTKKFKPLLFYDTRINQTTPIDTHPTFIWKNHRLPHDIPGLGP 432
SP|P25092|GUC2C_HUMAN          DDWGDVDSTMVLLYTSVDTKKYKVLLTYDTHVNKTYPVDMSPTFTWKNSKLPNDITGRGP 429
TR|F7B751|F7B751_MONDO         DEWGDIDNNVTILYTSADTTKYEVLLYYDTHRNETMWSKERPELNWKNHKLPSDEQGKDP 304
                               *  **:*..: :****  ..*:: *: ***: * :      * : * : :** *  . .

TR|A0A3Q2L9C5|A0A3Q2L9C5_HORSE QILMIAVFTLAGTIILLLLIALLVLRKYKKENELRQKKWSHIPPEKIFPLETNETNHISL 375
TR|A0A452E207|A0A452E207_CAPHI QILMIAVFTLAGTVVLLLLIALLVLRKYKREHALRQKKWSHIPPEDIFPLESNETNHVSL 467
TR|A0A2K5TZ15|A0A2K5TZ15_MACFA QILMIAVFTLTGAVVLLLLVALLMLRKYKKDYELRQKKWSHIPPENIFPLETNETNHVSL 449
TR|G3R542|G3R542_GORGO         QILMIAVFTLTGAVVLLLLVALLMLRKYRKDYELRQKKWSHIPPENIFPLETNETNHVSL 489
TR|G1LVN9|G1LVN9_AILME         QALLIAVFTLTGAVVLLLLIALLVLRKYKKEYELRQKKWSHIPPENIFPLETNETNHISL 493
TR|F1N5B2|F1N5B2_BOVIN         QMLMIAVFTLAGTVVLLLLIALLVLRKYKREHALRQKKWSHIPPEDIFPLESNETNHVSL 488
TR|A0A3Q7S8U5|A0A3Q7S8U5_VULVU QILLIAVFTLTGAVVLLLLIALLVLRKYKKEYELRQKKWSHIPPENIFPLETNETNHISL 532
TR|F6W2A3|F6W2A3_ORNAN         PFLMIVTFALTGAVFLILLIALLMIWKYRRDHELRQKKWSHIPSEKILALETNESNHISL 490
TR|H0VCJ2|H0VCJ2_CAVPO         HILLIAVCTLAGVVVLILLIALLVLRKYKKDNELRQKKWSHIPPEKILPLETNETNHVSL 492
SP|P25092|GUC2C_HUMAN          QILMIAVFTLTGAVVLLLLVALLMLRKYRKDYELRQKKWSHIPPENIFPLETNETNHVSL 489
TR|F7B751|F7B751_MONDO         EILLIAVFTLTGAVILILVISLLILRKNKKNNELRQKKWSHIPTEKIILLETNETGHVSL 364
                                 *:*.. :*:*.:.*:*:::**:: * :::  ********** *.*: **:**:.*:**

TR|A0A3Q2L9C5|A0A3Q2L9C5_HORSE KIDDDKRRDTIQRLQQCKYDKKRVILKDLKHNDGNFTEKQKIELNKLLQIDYYNLTKFYG 435
TR|A0A452E207|A0A452E207_CAPHI KIDEDKRRDTIQRLRQCKYDKKRVILKDLKHNDGNFTEKQKIELNKLLQIDYYNLTKFYG 527
TR|A0A2K5TZ15|A0A2K5TZ15_MACFA KIDDDKRRDTIQRLRQCKYDKKRVILKDLKHNDGNFTEKQKIELNKLLQIDYYNLTKFYG 509
TR|G3R542|G3R542_GORGO         KIDDDKRRDTIQRLRQCKYDKKRVILKDLKHNDGNFTEKQKIELNKLLQIDYYNLTKFYG 549
TR|G1LVN9|G1LVN9_AILME         KIDDDKRRDTIQRLRQCKYDKKRVILKDLKHNDGNFTEKQKIELNKLLQIDYYNLTKFYG 553
TR|F1N5B2|F1N5B2_BOVIN         KIDDDKRRDTIQRLRQCKYDKKRVILKDLKHNDGNFTEKQKIELNKLLQIDYYNLTKFYG 548
TR|A0A3Q7S8U5|A0A3Q7S8U5_VULVU KIDDDKRRDTIQRLRQCKYDKKRVILKDLKHNDGNFTEKQKIELNKLLQIDYYNLTKFYG 592
TR|F6W2A3|F6W2A3_ORNAN         KIDDDKRRDTGPRLRQGKYDKKIVILKDLKYNDGNFSEKQKIALNKLLQVDYYNLTKFYG 550
TR|H0VCJ2|H0VCJ2_CAVPO         KIDDDKKRDTVQRLRQCKYDQKRVILKDLKYSDGNFSEKQKIELNKLLQSDFYNLTKFYG 552
SP|P25092|GUC2C_HUMAN          KIDDDKRRDTIQRLRQCKYDKKRVILKDLKHNDGNFTEKQKIELNKLLQIDYYNLTKFYG 549
TR|F7B751|F7B751_MONDO         KIEEDKRRDTITRLRQGKYDKKGVILKALKNNDGNFTERQKIDLNKLLQIDYYNLTKFYG 424
                               **::**:***  **:* ***:* **** ** .****:*:*** ****** *:********

TR|A0A3Q2L9C5|A0A3Q2L9C5_HORSE TVKLDTTIFGVIEYCERGSLR---EVLNDTISYPDGTFMDWEFKISVLYDIAKGMSYLHS 492
TR|A0A452E207|A0A452E207_CAPHI TVKLDSMIFGVIEYCERGSLR---EVLNDTISYPDGTFMDWEFKISVLYDIAKGMSYLHS 584
TR|A0A2K5TZ15|A0A2K5TZ15_MACFA TVKLDTMIFGVIEYCERGSLR---EVLNDTISYPDGTFMDWEFKISVLYDIAKGMSYLHS 566
TR|G3R542|G3R542_GORGO         TVKLDTMIFGVIEYCERGSLR---EVLNDTISYPDGTFMDWEFKISVLYDIAK------- 599
TR|G1LVN9|G1LVN9_AILME         TVKLDTMIFGVIEYCERGSLR---EVLNDTISYPDGTFMDWEFKISVLYDIAKGMSYLHS 610
TR|F1N5B2|F1N5B2_BOVIN         TVKLDSTIFGVTEYCERGSLR---EVLNDTISYPDGTFMDWEFKISVLYDIAKGMSYLHS 605
TR|A0A3Q7S8U5|A0A3Q7S8U5_VULVU TVKLDTMIFGVIEYCERGSLR---EVLNDTISYPDGTFMDWEFKISVLYDIAKGMSYLHS 649
TR|F6W2A3|F6W2A3_ORNAN         TVKIDATIFGVIEYCERGSLR---DVLNDSISYPDGAFMDLEFKISVMYDIAKGMSYLHA 607
TR|H0VCJ2|H0VCJ2_CAVPO         TVKLDTRIFGVIEYCERGSLRLRNCVLNDTISYPDGTFMDWEFKISVLYDIAKGMSYLHS 612
SP|P25092|GUC2C_HUMAN          TVKLDTMIFGVIEYCERGSLR---EVLNDTISYPDGTFMDWEFKISVLYDIAKGMSYLHS 606
TR|F7B751|F7B751_MONDO         TVKIDTMIFGVIEYCERGSLR---EVLNDTISYPDGTFMDWEFKISVMYDIAKGMSYLHS 481
                               ***:*: **** *********    ****:******:*** ******:*****

TR|A0A3Q2L9C5|A0A3Q2L9C5_HORSE SKIEVHGRLKSTSCVVDSRMVVKITNFGCNSILPPRKDLWTAPEHLRHASFSQKGDVYSY 552
TR|A0A452E207|A0A452E207_CAPHI SKTEVHGRLKSTNCVVDSRMVVKITDFGCNSILPPRKDLWTAPEHLRVASVSQKGDVYSY 644
TR|A0A2K5TZ15|A0A2K5TZ15_MACFA SKTEVHGRLKSTNCVVDSRMVVKITDFGCNSILPPKKDLWTAPEHLRQANVSQKGDVYSY 626
TR|G3R542|G3R542_GORGO         ------------------------------------------------------------
TR|G1LVN9|G1LVN9_AILME         SKTEVHGRLKSTNCVVDSRMVVKITDFGCNSILPPKKDLWTAPEHLRQASISQKGDVYSY 670
TR|F1N5B2|F1N5B2_BOVIN         SKTEVHGRLKSTNCVVDSRMVVKITDFGCNSILPPRKDLWTAPEHLRVASVSQKGDVYSY 665
TR|A0A3Q7S8U5|A0A3Q7S8U5_VULVU SKTEVHGRLKSTNCVVDSRMVVKITDFGCNSILPPKKDLWTAPEHLRQANISQKGDVYSY 709
TR|F6W2A3|F6W2A3_ORNAN         SKTEVHGRLKSTNCVVDSRMVVKITDFGCSSILPPKKDLWTAPEHLRRASESQKGDVYSY 667
TR|H0VCJ2|H0VCJ2_CAVPO         SKIEVHGRLKSTNCVVDSRMVVKITDFGYNSILPPKKDLWTAPEHLRQASTSQKGDVYSF 672
SP|P25092|GUC2C_HUMAN          SKTEVHGRLKSTNCVVDSRMVVKITDFGCNSILPPKKDLWTAPEHLRQANISQKGDVYSY 666
TR|F7B751|F7B751_MONDO         SKTEVHGRLKSTNCVVDSRMVVKITDFGCNSILPTKKDLWMAPEHLREASVSQKGDVYSY 541
```

```
TR|A0A3Q2L9C5|A0A3Q2L9C5_HORSE GIIAQEIILRRETFYTSSCRDQNEKIFRVENSNGVKPFRPDLFLETAEEKELEVYLLVKN 612
TR|A0A452E207|A0A452E207_CAPHI GIIAQEIIQRRETFYTLSCRDQKEKIFRVENSNGVKPFRPDLFLETAEEKELEVYLLVKS 704
TR|A0A2K5TZ15|A0A2K5TZ15_MACFA GIIAQEIILRKETFYTSSCRDRNEKIFRVENSNGMKPFRPDLFLETAEEKELEVYLLVKS 686
TR|G3R542|G3R542_GORGO         -----------------------------------------------------VYLLVKN 606
TR|G1LVN9|G1LVN9_AILME         GVIAQEIILRRETFYTLSCRDQNEKIFRVENANGVKPFRPDLFLETAEEKELEVYLLVKN 730
TR|F1N5B2|F1N5B2_BOVIN         GIIAQEIIQRRETFYTLSCRDQKEKIFRVENCNGVKPFRPDLFLETAEEKELEVYLLVKS 725
TR|A0A3Q7S8U5|A0A3Q7S8U5_VULVU GIIAQEIILRRETFYTLSCRDQNEKIFRVENANGVKPFRPDLFLETAEEKELEVYLLVKN 769
TR|F6W2A3|F6W2A3_ORNAN         GIIAQEIILRKETFHTLCCWDPKEKIYRVEKAEGSKPFRPDLFLENAEEKEVEVYLLVKS 727
TR|H0VCJ2|H0VCJ2_CAVPO         GIIAQEIIMRRETFYTLSCRDQKEKIFRVEHPDGLKPFRPDLFLETAEEKELEVYLLVKN 732
SP|P25092|GUC2C_HUMAN          GIIAQEIILRKETFYTLSCRDRNEKIFRVENSNGMKPFRPDLFLETAEEKELEVYLLVKN 726
TR|F7B751|F7B751_MONDO         GIITQEIILRRETFYTLRCRDQKEKIFRVENYNGLKPFRPDLFLETTDEKELEVFLLVKN 601
                                                                                    *:****.

TR|A0A3Q2L9C5|A0A3Q2L9C5_HORSE CWEEDPEKRPDFKKIENTLAKIFGLFHDQK-NESYMDTLIRRLQLYSRNLEHLVEERTQL 671
TR|A0A452E207|A0A452E207_CAPHI CWEEDPEKRPDFKKIENTLAKIFGLFHDQK-NESYMDTLIRRLQLYSRNLEHLVEERTQL 763
TR|A0A2K5TZ15|A0A2K5TZ15_MACFA CWEEDPEKRPDFKKIETTLAKIFGLFHDQK-NESYMDTLIRRLQLYSRNLEHLVEERTQL 745
TR|G3R542|G3R542_GORGO         CWEEDPEKRPDFKKIETTLAKIFGLFHDQK-NESYMDTLIRRLQLYSRNLEHLVEERTQL 665
TR|G1LVN9|G1LVN9_AILME         CWEEDPEKRPDFKKIESTLAKIFGLFHDQK-NESYMDTLIRRLQLYSRNLEHLVEERTQL 789
TR|F1N5B2|F1N5B2_BOVIN         CWEEDPEKRPDFKKIENTLAKIFGLFHDQK-NESYMDTLIRRLQLYSRNLEHLVEERTQL 784
TR|A0A3Q7S8U5|A0A3Q7S8U5_VULVU CWEEDPEKRPDFKKIESTLAKIFGLFHDQK-SESYMDTLIRRLQLYSRNLEHLVEERTQL 828
TR|F6W2A3|F6W2A3_ORNAN         CWEEDPEKRPDFKKIESTLAKLYGNFHSQN-SESYMDTLIRRLQLYSRNLEHLVEERTEL 786
TR|H0VCJ2|H0VCJ2_CAVPO         CWEEDPEKRPDFKKIENTLAKIFGLFHDQK-NESYMDTLIRRLQLYSRNLEHLVEERTQL 791
SP|P25092|GUC2C_HUMAN          CWEEDPEKRPDFKKIETTLAKIFGLFHDQK-NESYMDTLIRRLQLYSRNLEHLVEERTQL 785
TR|F7B751|F7B751_MONDO         CWEEDPEKRPDFKKIESTLAKLFSHFHSQNNNESYMDSLIRRLQLYSRNLEHLVEERTKL 661
                               ***************.****::. **.*: .*****:*********************:*

TR|A0A3Q2L9C5|A0A3Q2L9C5_HORSE YKAERDRADRLNFMLLPRLVVKSLKEKGFVEPELYEEVTIYFSDIVGFTTICKYSTPMEV 731
TR|A0A452E207|A0A452E207_CAPHI YKAERDRADQLNFMLLPRLVVKSLKEKGFVEPELYEEVTVYFSDIVGFTTICKYSTPMEV 823
TR|A0A2K5TZ15|A0A2K5TZ15_MACFA YKAERDRADRLNFMLLPRLVVKSLKEKGFVEPELYEEVTIYFSDIVGFTTICKYSTPMEV 805
TR|G3R542|G3R542_GORGO         YKAERDRADRLNFMLLPRLVVKSLKEKGFVEPELYEEVTIYFSDIVGFTTICKYSTPMEV 725
TR|G1LVN9|G1LVN9_AILME         YKAERDRADRLNFMLLPRLVVKSLKEKGIVEPELYEEVTIYFSDIVGFTTICKYSTPMEV 849
TR|F1N5B2|F1N5B2_BOVIN         YKAERDRADQLNFMLLPRLVVKSLKEKGFVEPELYEEVTVYFSDIVGFTTICKYSTPMEV 844
TR|A0A3Q7S8U5|A0A3Q7S8U5_VULVU YKAERDRADRLNFMLLPRLVVKSLKEKGIVEPELYEEVTIYFSDIVGFTTICKYSTPMEV 888
TR|F6W2A3|F6W2A3_ORNAN         YKAERDRADRLNFMLLPRLVVKSLKETGLVEPELYEEVTIYFSDIVGFTTICKYSTPMEV 846
TR|H0VCJ2|H0VCJ2_CAVPO         YKAERDRADRLNFMLLPRPVVQSLKEKGIVEPELYEEVTVYFSDIVGFTTICKYSTPMEV 851
SP|P25092|GUC2C_HUMAN          YKAERDRADRLNFMLLPRLVVKSLKEKGFVEPELYEEVTIYFSDIVGFTTICKYSTPMEV 845
TR|F7B751|F7B751_MONDO         YKAERDRADRLNFMLLPRLVVKSLKEKGFVEPELYEEVTIYFSDIVGFTTICKYSTPMEV 721
                               *********:******** **:****.*:**********:********************

TR|A0A3Q2L9C5|A0A3Q2L9C5_HORSE VDMLNDIYKSFDHILDHHDVYKVETIGDAYMVASGLPKRNGNRHAVDIAKMALDILSFMG 791
TR|A0A452E207|A0A452E207_CAPHI VDMLNDIYKNFDHILDHHDVYKVETIGDAYMVASGLPKRNGNRHAIDIAKMALDLLSFVG 883
TR|A0A2K5TZ15|A0A2K5TZ15_MACFA VDMLNDIYKSFDHIVDHHDVYKVETIGDAYMVASGLPKRNGNRHAIDIAKMALEILSFMG 865
TR|G3R542|G3R542_GORGO         VDMLNDIYKSFDHIVDHHDVYKVETIGDAYMVASGLPKRNGNRHAIDIAKMALEILSFMG 785
TR|G1LVN9|G1LVN9_AILME         VDMLNDIYKSFDHILDHHDVYKVETIGDAYMVASGLPKRNGNRHAIDIAKMALDILSFMG 909
TR|F1N5B2|F1N5B2_BOVIN         VDMLNDIYKNFDHILDHHDVYKVETIGDAYMVASGLPKRNGNRHAIDIAKMALDLLSFVG 904
TR|A0A3Q7S8U5|A0A3Q7S8U5_VULVU VDMLNDIYKSFDHILDHHDVYKVETIGDAYMVASGLPKRNGNRHAIDIAKMALDILSFMG 948
TR|F6W2A3|F6W2A3_ORNAN         VDMLNDVYMSFDHILDHHDVYKVETIGDAYMVASGLPKRNGHRHAVDIAKMALDILSFMG 906
TR|H0VCJ2|H0VCJ2_CAVPO         VDMLNDLYKSFDQIVDHHDVYKVETIGDAYVVASGLPTRNGNRHAIDISKMALDILSFIG 911
SP|P25092|GUC2C_HUMAN          VDMLNDIYKSFDHIVDHHDVYKVETIGDAYMVASGLPKRNGNRHAIDIAKMALEILSFMG 905
TR|F7B751|F7B751_MONDO         VDMLNDIYKSFDHILDHHDVYKVETIGDAYMVASGLPERNGNRHAVDIAKMALDILSFMG 781
                               ******:* .**:*:***************:****** ***:***:**:****::***:*

TR|A0A3Q2L9C5|A0A3Q2L9C5_HORSE TFELEHLPDLPIWIRIGVHSGPCAAGVVGIKMPRYCLFGDTVNTASRMESTGLPLRIHVS 851
TR|A0A452E207|A0A452E207_CAPHI TFELEHLPGLPIWIRIGIHSGPCAAGVVGIKMPRYCLFGDTVNTASRMESTGLPLRIHVS 943
TR|A0A2K5TZ15|A0A2K5TZ15_MACFA TFELEHLPGLPIWIRIGVHSGPCAAGVVGIKMPRYCLFGDTVNTASRMESTGLPLRIHVS 925
TR|G3R542|G3R542_GORGO         TFELEHLPGLPIWIRIGVHSGPCAAGVVGIKMPRYCLFGDTVNTASRMESTGLPLRIHVS 845
TR|G1LVN9|G1LVN9_AILME         TFQLEHLPGLPIWIRIGVHSGPCAAGVVGIKMPRYCLFGDTVNTASRMESTGLPLRIHVS 969
TR|F1N5B2|F1N5B2_BOVIN         TFELEHLPGLPIWIRIGIHSGPCAAGVVGIKMPRYCLFGDTVNTASRMESTGLPLRIHVS 964
TR|A0A3Q7S8U5|A0A3Q7S8U5_VULVU TFELEHLPGLPIWIRIGVHSGPCAAGVVGIKMPRYCLFGDTVNTASRMESTGLPLRIHVS 1008
TR|F6W2A3|F6W2A3_ORNAN         SFELRHLPGLPVWIRIGVHSGPCAAGVVGIKMPRYCLFGDTVNTASRMESTGLPLRIHVS 966
TR|H0VCJ2|H0VCJ2_CAVPO         TFELEHLPGLPVWIRIGVHSGPCAAGVVGIKMPRYCLFGDTVNTASRMESTGLPLRIHMS 971
SP|P25092|GUC2C_HUMAN          TFELEHLPGLPIWIRIGVHSGPCAAGVVGIKMPRYCLFGDTVNTASRMESTGLPLRIHVS 965
TR|F7B751|F7B751_MONDO         TFELQHLPGLPVWIRIGVHSGPCAAGVVGIKMPRYCLFGDTVNTASRMESTGLPLRIHVS 841
                               :*:*.***.**:*****:****************************************:*

TR|A0A3Q2L9C5|A0A3Q2L9C5_HORSE GSTISILKRTECQFLYEVRGETYLKGRGTETTYWLTGVKDQEYNLPTPPTVENQQRLQAE 911
TR|A0A452E207|A0A452E207_CAPHI GSTIAILKRTECQFLYEVRGETYLKGRGTETTYWLTGVKDQEYNLPTPPTAENQQRLQAE 1003
TR|A0A2K5TZ15|A0A2K5TZ15_MACFA GSTIAILKRTECQFLYEVRGETYLKGRGNETTYWLTGMKDQKFNLPTPPTVENQQRLQAE 985
TR|G3R542|G3R542_GORGO         GSTIAILKRTECQFLYEVRGETYLKGRGNETTYWLTGMKDQKFNLPTPPTVENQQRLQAE 905
TR|G1LVN9|G1LVN9_AILME         GSTIAILKRTECQFLYEVRGETYLKGRGTETTYWLTGVKGQEYTLPIPPTVENQQRLQAE 1029
TR|F1N5B2|F1N5B2_BOVIN         GSTIAILKRTECQFLYEVRGETYLKGRGTETTYWLTGVKDQEYNLPTPPTAENQQRLQAE 1024
TR|A0A3Q7S8U5|A0A3Q7S8U5_VULVU GSTIAILKRTECQFLYEVRGETYLKGRGTETTYWLTGVKGQEYNLPTPPTVENQQRLQAE 1068
TR|F6W2A3|F6W2A3_ORNAN         GSTIAILKRTQCQFLYEVRGETFLKGKGTETTFWLTGMKHQEYNLPTPPSVENQQRLQAE 1026
TR|H0VCJ2|H0VCJ2_CAVPO         SSTIAILKRVQCQFLYEMRGETYLKGKGTETTYWLTGMKDQEYNLPTPPTVENQQRLQAE 1031
SP|P25092|GUC2C_HUMAN          GSTIAILKRTECQFLYEVRGETYLKGRGNETTYWLTGMKDQKFNLPTPPTVENQQRLQAE 1025
TR|F7B751|F7B751_MONDO         GSTIAILKRTECQFLYEVRGETYLKGRGTETTYWLTGVKEQEYTLPTPPSVENQQHLQAE 901
                               .***:****.:******:****:***:*.***:****:* *::.** **:.****:****
```

```
TR|A0A3Q2L9C5|A0A3Q2L9C5_HORSE FSDMIANSLQKRQASGIRSRKPTRIASYKKGTLEYLQLDTTDQESTHF- 959
TR|A0A452E207|A0A452E207_CAPHI FADMIASSLQKRQALGIRNRKPTRVASYKKGTLEYLQLNTTDNESTHF- 1051
TR|A0A2K5TZ15|A0A2K5TZ15_MACFA FSDMIANSLQKRQAAGIRSQKPRRVASYKKGTLEYLQLNTTDKESTYF- 1033
TR|G3R542|G3R542_GORGO         FSDMIANSLQKRQAAGIRSQKPRRVASYKKGTLEYLQLNTTDKESTYF- 953
TR|G1LVN9|G1LVN9_AILME         FSDMLANSLQKRQAAGIRSRKPTRVASYKRGTLEYLQLNTTDKENTQV- 1077
TR|F1N5B2|F1N5B2_BOVIN         FADMIASSLQKRQALGIRNRKPTRVASYKKGTLEYLQLNTTDNESTHF- 1072
TR|A0A3Q7S8U5|A0A3Q7S8U5_VULVU FSDMLANSLQKRQAAGIRSRKPTRVASYKKGTLEYLQLNTTDKEIPPAN 1117
TR|F6W2A3|F6W2A3_ORNAN         FSNMITESLRKRQAEGFKRQKPSRVASYREGSLQYLQVATPDHSSGTYL 1075
TR|H0VCJ2|H0VCJ2_CAVPO         FSDMITNSLQKRQATGIKSRKPARVASYKKGTLEYLQLNTTDQDSTYF- 1079
SP|P25092|GUC2C_HUMAN          FSDMIANSLQKRQAAGIRSQKPRRVASYKKGTLEYLQLNTTDKESTYF- 1073
TR|F7B751|F7B751_MONDO         FSNMIVESLQKRQAQGLANQKPRRVASYKKGSLEYLHLNTTNDNNTYF- 949
                               *::*:..**:**** *:  :** *:***:.*:*:**:: * :..
```

Suppemental figure 1. Alignment of guanylate cyclase protein sequences using ClustalW2. Highlighted areas indicate: ■ Signal peptide, ■ Guanylate cyclase domain, ■ Protein kinase domain. Asterisk (*) indicate sequence identity, colon (:) indicates strongly similar properties and period (.) indicates weakly similar properties.
