# Supplementary figures and images for: Guanylin, Uroguanylin and Guanylate Cyclase-C Are Expressed in the Gastrointestinal Tract of Horses
Source: Front Physiol. 2019 Sep 27;10:1237. doi: 10.3389/fphys.2019.01237 (PMC6776823; doi:10.3389/fphys.2019.01237)

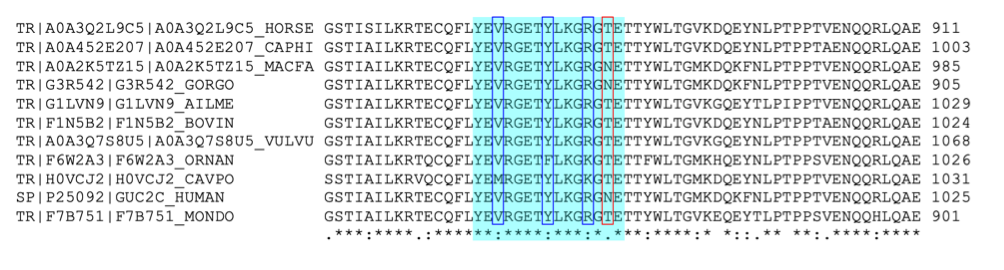

Supplement: FIGURE S2 — Epitope sequences alignment on ClustalW of several species. [file Image_2.png]
